# Injury Risk Factors in a Small-Scale Gold Mining Community in Ghana’s Upper East Region

**DOI:** 10.3390/ijerph120808744

**Published:** 2015-07-24

**Authors:** Rachel N. Long, Kan Sun, Richard L. Neitzel

**Affiliations:** 1 Department of Environmental Health Sciences, University of Michigan School of Public Health, Ann Arbor, MI 48109, USA; E-Mails: rachlong@umich.edu (R.N.L.); kansun@umich.edu (K.S.); 2 Risk Science Center, University of Michigan School of Public Health, Ann Arbor, MI 48109, USA

**Keywords:** small-scale gold mining, ASGM, injury risk, Ghana, occupational health

## Abstract

Occupational injury is one of many health concerns related to small-scale gold mining (ASGM), but few data exist on the subject, especially in sub-Saharan Africa. In 2011 and 2013, we examined accidents, injuries, and potential risk factors in a Ghanaian ASGM community. In 2011, 173 participants were surveyed on occupational history and health, and 22 of these were surveyed again in 2013. Injury rates were estimated at 45.5 and 38.5 injuries per 100 person-years in 2011 and in 2013, respectively; these rates far surpass those of industrialized mines in the U.S. and South Africa. Demographic and job characteristics generally were not predictive of injury risk, though there was a significant positive association with injury risk for males and smokers. Legs and knees were the most common body parts injured, and falling was the most common cause of injury. The most common type of injuries were cuts or lacerations, burns and scalds, and contusions and abrasions. Only two miners had ever received any occupational safety training, and PPE use was low. Our results suggest that injuries should be a priority area for occupational health research in ASGM.

## 1. Introduction

Artisanal and small-scale mining (ASM) is proliferating worldwide owing to increasing demand and rising prices for minerals and precious metals in growing economies [1]. At least 20 million people work directly in ASM, and 80–100 million are dependent on it for their livelihood [1]. In Ghana, an important gold-producing country, artisanal and small-scale gold mining (ASGM) accounted for 10.5% of Ghana’s national gold production and employed 0.5–1 million people as of 2010 [2,3]. Gold production from ASGM in Ghana rose 43% from 2011–12 [4]. Approximately 70% of mining operations in Ghana are illegal; that is, they are not registered and licensed under the provisions of the Small-Scale Gold Mining Act of 1999 [5].

The ASGM process begins with the excavation of gold-containing ore, which can be accomplished by panning in streams, surface and shallow digging, or by tunneling using shovels, picks, heavy excavation equipment, or dynamite. Ore is then crushed using a large hand-held mortar and pestle or generator-powered grinding machines, or both. Once a fine powder is produced, it is sifted through cloth over a basin. Large pieces trapped in the cloth are re-ground and re-sifted. This fine ore is mixed with water and carefully poured down a carpeted ramp. The carpet traps dense gold-containing particles, and less dense particles are washed down the ramp. The trapped particles are added to a pan with water, and gently centrifuged by hand to further separate the gold from the less dense ore. The gold particles are then mixed in small volumes with several drops of mercury, which forms an amalgam with the gold. Miners heat the amalgam with a blowtorch or small fire, which volatilizes the mercury and leaves behind a piece of concentrated gold. At this stage, the gold is usually sold to dealers, who repeat the mercury amalgamation and volatilization process to further refine the gold. 

ASGM processes pose many occupational and community health hazards. The most well-documented of these is mercury exposure [6,7], though ASG miners and communities are also potentially exposed to other heavy metals present in the gold ore, airborne silica dust from pulverized ore, and noise from mining equipment [8,9,10,11]. In addition to these exposures, hazardous physical conditions and activities throughout the process raise concerns about occupational injuries. Even in high-income countries with strong and well-enforced occupational health and safety regulations, mining is a hazardous activity. Rock falls, fires, explosions, mobile equipment accidents, falls, entrapment, and electrocution are all common causes of injury in mining [12]. The lack of safety regulations and enforcement, education and training, and functional infrastructure and equipment may lead to elevated injury rates in low- and middle-income countries, particularly in ASM. ASM operations may have 6–7 times more non-fatal accidents than large-scale operations [13], but only a few studies have documented occupational safety issues at ASGM sites in Ghana [14,15]. In a study in Ghana's Kumasi Basin, illegal ASG miners identified life-threatening risks at the mine site, including dynamite blasts, collapsing pits and shafts, falls near excavation sites. The respondents reported these risks more often than risks posed by mercury use [16]. 

While engineering controls are the preferred approach to mitigate safety hazards, in resource-limited settings such approaches are not often adopted. Unfortunately, the less-preferred alternative to reduce occupational risks, use of personal protective equipment (PPE, e.g., hardhats, safety glasses, gloves, work boots, *etc.*), is also uncommon [13,17]. In a survey of ASG miners in Ghana’s Upper East Region, 70% responded that they never use PPE [7]. In Wassa West District, Ghana, <13% of ASG miners wore safety boots, gloves, or a helmet while working [18]. 

While there are numerous injury risk factors associated with ASGM work, the frequency and nature of injuries have not been well documented in sub-Saharan Africa, where there are no systematic injury surveillance programs. Because of the informal and often illegal status of many ASG operations, injuries are underreported. In 1999, the ILO estimated that 5–20 fatalities occurred in Ghanaian small-scale mines, but the true number may be much higher, especially given the increase in mining activities in Ghana in recent years [13]. What data do exist from sub-Saharan Africa are from site-specific studies, and few calculate injury rates. In Ghana’s Wassa West District, 46.6% of small-scale gold miners surveyed reported at least one occupational injury in the last 10 years [18]. In the Democratic Republic of the Congo, 72% of small-scale miners surveyed had experienced at least one accident in the prior year, with most of these accidents being caused by tools and the handling of heavy loads [19]. In a study in Geita District in Tanzania, mining accidents caused about 11 fatalities annually, mostly resulting from tunnel collapse or exposure to toxic gases [20]. 

In 2012, an online search of Ghanaian newspaper articles resulted in 19 articles reporting 23 separate incidents of accidents and injuries among ASG miners occurring between 2007–12 [21]. Eight main incident types were described, with the most common being collapses trapping miners (30%), followed by drowning (17%) and violent incidents and falls into mine pits (13% each). Crushing, burns, suffocation, and firearms injuries were among the other types of incidents reported. Most of the incidents (70%) took place in illegal AS mines, and the vast majority (87%) of the reported injuries resulted in fatalities. Seventy-six fatalities were recorded in total, with a range of 1–18 fatalities per incident. While these data have potentially substantial reporting biases, this study does provide some insight into the nature of injuries in Ghanaian ASGM sites.

To better characterize injuries in ASGM sites in Ghana, our team collected cross-sectional and longitudinal data on accidents, injuries, and potentially related risk factors in Kejetia, a legally registered ASGM community in the Talensi District in Ghana's Upper East Region, where our research team has conducted research since 2009.

## 2. Methods

### 2.1. Data Collection

Data for this study were collected in Kejetia May–July 2011 and again in May 2013. Institutional Review Board (IRB) approval was obtained through the University of Michigan (HUM00028444 and HUM00073615). The community’s traditional chief and local concession owners gave us permission to work within the community.

Kejetia’s economy is centered around small-scale gold mining, and other enterprises such as beer parlors, food stands, seamstress’ shops, and bicycle repair services have emerged to meet demand for goods and services. Kejetia lacks maps, population estimates, or clear administrative boundaries. In order to achieve random sampling, in 2011, a handheld global positioning system unit (Oregon 450, Garmin International, Inc., Olathe, KS, USA) was used to take a set of geographic coordinates at every household structure in Kejetia. Households were defined as a group of people who eat from the same “pot,” in congruence with local cultural definitions. The community was then geographically divided into 20 clusters of approximately 20 households each, and each household in a cluster was assigned a unique number. Households were selected for interviewing by randomly pulling numbers from a bag, and up to three households were interviewed per day, each from different clusters. By the end of the sampling period, two to three households from each cluster had participated. When a household declined participation, another number from within that cluster was drawn from the bag. Less than 15% of households refused participation, and most cited lack of time as the main reason. Sampling in this manner occurred for six weeks due to funding limitations. 

US university students administered surveys written in English with the assistance of local Ghanaian translators, who verbally translated the questions into the participants' preferred language (Talen, Nabt, Gurune, Twi, Dagbani, English, or Hausa). Before conducting interviews, the translators were trained in the necessary medical vocabulary. In 2011, interviews were preferentially administered to the head of household (HOH), followed by their spouse or any adult (age 18 or older) who could knowledgeably answer questions about the other individuals in the household. This primary interviewee completed a survey on demographics (including age, sex, educational history, health, smoking status, and marital status) and familial relationships among household members. Four adults per household at a maximum (including the primary interviewee) were administered a separate occupational history survey, which included questions regarding individuals’ experience in a range of mining activities (excavation, crushing, sifting, washing, amalgamation, burning, and owning or managing a mine site). The HOH or other primary interviewee advised the researchers whom to interview in households with more than four interested adults.

In 2013, participants from 2011 were recruited via convenience sampling. Household GPS coordinates taken in 2011 were used to relocate households of all former participants who reported involvement in mining in the 2011 survey. Any willing former participant who could be identified in this manner was invited to be surveyed. Participants administered a questionnaire examining demographics (including age, sex, educational history, health, smoking status, and marital status) and occupational health and safety. The survey included questions on psychological job demands and co-worker support from the Job Content Questionnaire [22], stress-related questions from Cohen’s Perceived Stress Scale (PSS) [23], and injury history (total number of injuries and, for the worst injury, task at time of injury, cause of injury, type of injury, body part injured, medical attention received, and missed work). No threshold of severity was defined for injuries, thus even minor injuries were counted. To facilitate translation, we used dichotic questions (0 = No, 1 = Yes) for the five psychological job demands questions and the four coworker support questions instead of the conventional four-point Likert scales for these scales. For analysis, we then converted the dichotic responses to the four-point Likert scale by replacing “No” with 1 and “Yes” with 4. We used five of the fourteen PSS questions and reported the summary score on a 0–20 scale, with higher scores indicating greater perceived work demands. We also summed the psychological demands and coworker support scales (scale of 4–16), with higher scores indicating greater demands and greater coworker support, respectively. Additional survey items on nutrition, as well as measurements of noise exposure, heart rate, and salivary cortisol, were collected in 2013 and are described in another paper [24].

### 2.2. Analysis

Data were entered into Microsoft Excel (Microsoft, 2010) and then transferred to SPSS 22 (IBM, Armonk, NY, USA) for analysis. Descriptive statistics were calculated by demographic and occupational factors. BMI was computed from height and weight information, and scores for the psychological job demands, co-worker support [22], and PSS [23] scales were computed. One-way ANOVA and independent sample t-tests were used to evaluate potential differences in continuous variables between the 2011 sample and 2013 sub-sample, and Chi-square and Fisher’s exact tests were used for categorical variables. We estimated injury rate per person-year by dividing the number of surveyed miners injured in a given year by the number of all surveyed ever-miners and multiplied that by 100, resulting in a rate expressed in people injured/100 person-years. We also adjusted this rate for the number of hours worked in mining per week during the week of the survey by dividing the number of hours worked per week by 40 hours, and multiplying the resulting fraction by the injury rate. In both 2011 and 2013, we excluded those who had not actively mined in the preceding three months. Finally, a binary logistic regression model was used to identify factors predictive of increased injury risk. Demographic factors (e.g., age, BMI, education, religion, ethnicity, marital status and education), mining activities (excavation, crushing/grinding, sifting/shanking, washing/sluicing, amalgamation, burning, and owning/managing a mine) and mining experience, hours worked per week, and frequency of PPE use (defined as apron, coverall, gloves, hat, helmet/hard hat, rubber soled shoes, safety boots/steel toed boots, or safety goggles) were tested individually and in combination for potential association with injury risk. Mining experience was estimated using the greatest number of years an individual had spent on any single mining activity. Independent variables were retained in the model where *p* < 0.05, and mining experience was forced into the model due to its potentially confounding role.

## 3. Results 

### 3.1. Demographics and Work Experience 

In 2011, 173 participants were recruited, of whom just under half were male (Table 1). Participants had an average age of 40.1 ± 17.8 years, and most (approximately 75%) were non-smokers and ever married. Overall mean BMI was 22.4 ± 3.2; 17.3% were overweight or obese and 9.80% were underweight. Nearly half (46.8%) had never received formal education and less than a quarter received education beyond middle school. 

About 76% of the households surveyed contained at least one current miner. About half of participants had never worked in mining, 8.1% previously worked in mining, and 41.6% were current miners. We classified the latter two categories collectively as "ever-miners." Of all the ever-miners, 51.2% reported they had ever excavated ore rocks, 61.6% had ever crushed rocks, 58.1% had ever sifted or washed/sluiced crushed rocks, 65.1% had ever amalgamated using mercury, and 43.0% had ever burned mercury. Only a quarter (25.6%) had ever owned or managed a mining site (Table 1). All but one were male. Significantly more male ever-miners had ever worked on excavating, crushing, washing, amalgamation and burning (Table S1). Overall, male ever-miners had significantly more years of mining experience than female (8.7 ± 6.7 yr *vs*. 4.5 ± 5.3 yrs), particularly in sifting, washing, amalgamation and burning (Table S1). Though more females had ever worked in sifting, on average they spent significantly fewer years in sifting than males. On average, ever-miners spent 36.9 ± 46.6 hours per week mining, and there was no significant difference between males and females (Table S1). Less than 10% of ever-miners reported ever wearing any of the common types of PPE (data not shown) and we observed that most miners wore t-shirts, long pants, and flip-flops or open rubber sandals during their work.

**Table 1:** Demographic and occupational information for 2011 (N = 173) sample and 2013 subsample (N = 22).

	2011					2013					
	Mean ± SD	n		%		Mean ± SD			n		%
***Demographics***											
**Age**	40.1 ± 17.8 *					34.1 ± 10.3					
≤24	-	35		20.2		-			4		18.2
25–34	-	48		27.7		-			8		36.4
35–44	-	32		18.5		-			6		27.3
≥45	-	58		33.5		-			4		18.2
**Male sex**		84		48.6					10		45.5
**BMI**	22.4 ± 3.2	-		-		22.5 ± 3.4			-		-
Underweight	-	17		9.80		-			3		13.6
Normal	-	126		72.8		-			15		68.2
Overweight/obese	-	30		17.3		-			4		18.1
**Ever smoked**	-	45		26.0		-			5		22.7
Ever Married **	-	152		87.9		-			18		81.8
**Highest education level**											
Never attended school	-	81		46.8		-			7		31.8
Primary or Less	-	51		29.5		-			7		31.8
Middle, Secondary or higher	-	40		23.0		-			8		36.3
Don’t know	-	1		0.6		-			0		0
**Religion**											
No religion	-	-		-		-			3		13.6
Christian	-	-		-		-			9		40.9
Muslim	-	-		-		-			4		18.2
Traditional	-	-		-		-			6		27.3

Occupational Information *											
Never-miner	-	87		50.3		-			5		22.7
Ever-miner											
Ex-miner		14		8.1		-			4		18.2
Current miner	-	72		41.6		-			13		59.1

**Ever-miners**	**2011 (N = 86)**							**2013 (N = 17)**			
	**n (%)**		**Yrs Experience (Mean ± SD)**					**n (%)**		**Yrs Experience (Mean ± SD)**	
Ever excavated	44 (51.2) **		7.7 ± 6.1					10 (58.8)		5.8 ± 6.1	
Ever crushed	53 (61.6) **		6.5 ± 6.3					11 (64.7)		5.5 ± 6.0	
Ever sifted	50 (58.1) *		5.8 ± 6.0					10 (58.8)		5.8 ± 5.3	
Ever washed	50 (58.1) ***		7.7 ± 6.8					13 (76.5)		7.9 ± 6.1	
Ever amalgamated	56 (65.1) ***		7.1 ± 6.7					14 (82.4)		8.5 ± 5.8	
Ever burned	37 (43.0) **		5.8 ± 5.4					9 (52.9)		5.3 ± 5.4	
Ever owned/managed mine	22 (25.6)		5.7 ± 5.7					5 (29.5)		2.6 ± 5.3	
Mining experience	-		6.9 ± 6.4					-		7.7 ± 5.8	
Total working hrs/wk	-		36.9 ± 46.6					-		31.0 ± 28.9	

Significant difference between 2011 and 2013: * *p* ≤ 0.05, ** *p* ≤ 0.01, *** *p* ≤ 0.005.

Because of the mining community's transient nature, only 22 of the 173 participants (10 males and 12 females) sampled in 2011 were available to participate in 2013 (Table 1). On average, they were 6 years younger (34.1 ± 10.3 yrs) than participants surveyed in 2011, but had similar distribution across age categories (*p* = 0.025, Table 1). The male to female ratio and BMI of the 2013 subsample was similar to 2013. Although there were more underweight and fewer normal weight participants in 2013, the difference in distribution was non-significant. There were no significant differences in smoking status or education level between the two years. Slightly but significantly fewer participants in 2013 had ever married. Most of the revisited participants were ever-miners. A slightly but significantly higher rate of ex-miners had ever worked in excavating, crushing, sifting, washing, amalgamation and burning. Similar to 2011, the majority of male ever-miners had worked in excavation, crushing, washing and amalgamation. Significantly more of them had ever crushed ore. Only 22.2% of males had ever worked in sifting compared to 100% of females (Table S1). Male ever-miners had more years of mining experience than females (8.6 ± 6.4 yrs *vs*. 6.8 ± 5.2 yrs), especially in amalgamation. More than a quarter of participants (29.5%), exclusively male, had ever owned/managed a mining site. On average, ex-miners from 2013 spent 31.0 ± 28.9 hours per week on mining. Their PPE use was consistently low. Only one participant had ever worn gloves, while 31.3% had ever worn a dust mask (data not shown). Only two participants from 2013 (both male) had ever received safety training. More than half (52.9%) reported that they had refused to do work because of personal safety concerns, and 80% had expressed their concerns or made complaints about their safety in mine. 

Among the 2013 subsample, the average psychological job demands scores of female and male ever-miners were significantly different (higher scores indicate greater psychological job demands) (data not shown). Female ever-miners had an average score of 24.5 ± 15.0, compared to 36.2 ± 9.4 for males. The average co-worker support scores for female and male ever-miners were 15.3 ± 1.6 and 14.7 ± 2.8, near the highest possible support score on the 16-point scale. PSS scores above half of the full scale (10 in this case) are considered “high stress”. Ever-miners had an average PSS score of nearly half of the full scale: 9.9 ± 2.1. Moreover, male ever-miners perceived slightly higher stress than females (data not shown).

### 3.2. Injuries in 2011 and 2013 

Of the 86 ever-miners in 2011, injury rates differed significantly by BMI category, sex, and smoking status (*p* < 0.005, data not shown). Among the 17 ever-miners interviewed in both 2011 and 2013, eight (valid percent 50.0%) were injured in 2011 and five (valid percent 38.5%) were injured in 2013 (Table 2). Injury risk was significantly associated with BMI in 2011 but not 2013. Male ever-miners were significant more likely to experience an injury in both years. There was no clear trend in numbers of injuries by age group or marital status in 2011 and 2013. Participants who received education higher than middle school and who adhered to traditional religions were more likely to be injured in both 2011 and 2013, though neither finding was significant. 

**Table 2:** Injury experience for 2013 subsample of ever-miners in 2011 and 2013, N = 17.

Variable	2011				2013		
	N Respondents	N injured	%		N Respondents	N injured	%
**Current occupation**							
Mining	16	8	50.0		13	5	38.5
**Age**							
≤24	3	3	100.0		2	1	50.0
25–34	7	3	42.9		6	1	16.7
35–44	4	1	25.0		3	2	66.7
≥45	2	1	50.0		2	1	50.0
**BMI**							
Underweight	3	0	0.0 *		3	0	0.0
Normal	15	5	33.3 *		15	5	33.3
Overweight/obese	4	0	0.0 *		4	2	50.0
**Sex**							
Male	8	6	75.0		9	5	55.6 *
Female	8	2	25.0		4	0	0.0 *
**Smoking status**							
Never smoked	12	6	50.0		9	2	22.2
Ever smoked	4	2	50.0		4	3	75.0
**Marital status**							
Never married	3	2	66.7		3	1	33.3
Ever married	13	6	46.2		10	4	40.0
**Highest education level**							
Never attended school	4	1	25.0		4	1	25.0
Primary or Less	5	2	40.0		3	0	0.0
Middle, Secondary or higher	7	5	71.4		6	4	66.7
**Religion**							
No religion	2	1	50.0		1	1	100.0
Christian	5	3	60.0		4	2	50.0
Muslim	3	2	66.7		2	1	50.0
Traditional	6	2	33.3		6	1	16.7

Difference of injury rate across each predictor category in either year * *p* ≤ 0.05.

**Figure 1:**
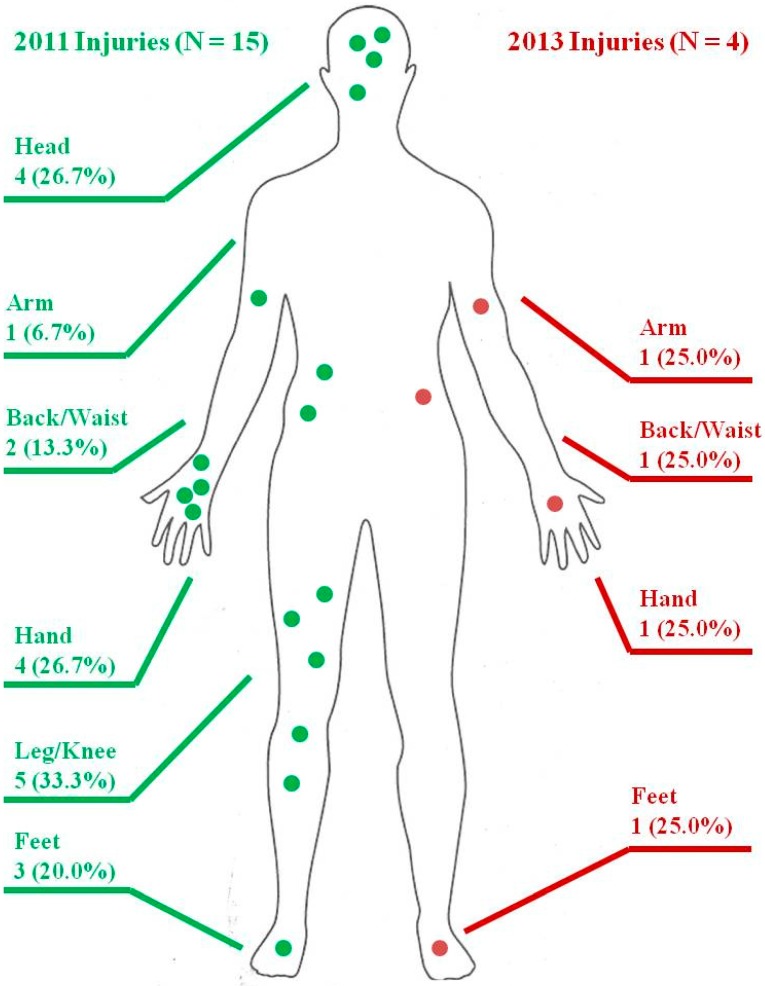
Body injury locations and frequencies for worst injuries in 2011* and 2013**. * Reported injured body part(s) from mining-related injuries in the past year among 86 ever-miners in2011. The information is based on an open-ended question adapted into a similar question in 2013 for comparison. Only 15 out of 35 injured participants provided information about injured body parts. ** Reported injured body part(s) from the worst injury in the past year among 17 ever-miners in 2013. 4 out of 5 injured participants provided information about injured body parts.

The dominant type of previous year injury in 2011 and 2013 was cuts and lacerations (Table 3). Reported injuries were distributed across all body parts in both years (Figure 1), with leg, knee, and foot injuries being predominant in 2011. Information on mining task during injury was available from participants in 2013 but not 2011 (Table 3). Falling accounted for 50% of previous-year injuries, and being struck or hit by an object and machinery and tools each accounted for 25%. None of the injured workers were wearing PPE at the time of injury. Three quarters of the injuries required no medical attention and only one (representing 25% of injuries) of the injured participants missed work for 7 days (Table 3). 

### 3.3. Miners Injured in Both 2011 and 2013 

Four out of the five miners that were injured in 2011 were also injured in 2013 (data not shown). They were all male. No systematic differences between these multiple-injury workers and the other 13 miners evaluated in both years were identified, though three had owned a concession or business and had >10 years of experience in ASGM, which was at the higher end among all ever-miners. The four miners had diverse experience in the six mining tasks assessed. Their psychological demands, coworker support, and perceived stress scores did not differ substantially from those of other participants. Two out of the four had refused to work because of personal safety concerns, and two reported ever wearing PPE in any form. The only two workers who had received prior safety training among all 17 ever-miners were injured in both years. 

**Table 3:** Summary of information on individual ever-miners’ injuries for 2011 (n = 86) and 2013 (n = 17).

Variable	2011 ^§^ (Total Injuries = 35)		2013 ^§§^ (Total Injuries = 5)	
	N	Valid %	N	Valid %
***Worst injury***				
**Tasks**			5	
Excavation	-	-	1	25.0
Crushing	-	-	2	50.0
Moving subject	-	-	-	-
Resting	-	-	1	25.0
Missing	-	-	1	
**Injury cause**			5	
Burn	-	-	-	-
Fall	-	-	2	50.0
Struck/hit by object	-	-	1	25.0
Machinery/tool	-	-	1	25.0
Missing	-	-	1	
**Medical attention**			5	
No medical care	-	-	2	50.0
Treatment in medical clinic or office	-	-	-	-
Treatment at hospital	-	-	1	25.0
Other	-	-	1	25.0
Missing	-	-	1	
**Missed work**			5	
No missed work; did regular work	-	-	3	75.0
No missed work but did not do regular work	-	-	-	-
Missed work	-	-	1	25.0
Missed work (# days)	-	-	7.0	
Missing	-	-	1	
***All injuries***				
**Type of injury**	35		6 ^§§§^	
Cut/laceration	14	43.8	3	60.0
Contusion and abrasion	7	21.9	1	20.0
Fall	1	3.1	-	-
Burn/scald	-		-	-
Non-specific (bruise or cut/bruise)	10	31.2	-	-
Internal pain	-	-	1	20.0
Missing	3		1	

^§^ Injury information from 2011 is based on an open-ended question regarding all mining related injuries in the past year. This was fitted into a similar question in 2013 for comparison. ^§§^ Injury information from 2013 focused only on worst injury in the past year. ^§§§^ Included multiple injury types per one sustained injury.

### 3.4. Injury Rates in 2011 and 2013

Thirty-five out of 77 (45.5%) ever-miners reported at least one injury in 2011, and the estimated past-year injury rate was 45.5 injuries per 100 person-years. Five ever-miners among the 13 ever-miners surveyed in 2013 had at least one injury in the past year, for an estimated past-year injury rate was 38.5 per 100 person-years. These estimates would likely be higher if multiple injuries per person were considered. Adjusting for the number of hours worked in mining in the week preceding the survey, the estimated past-year injury rates were similar but lower: 42.0 injuries per 100 person-years in 2011 and 29.9 injuries per 100 person-years in 2013.

### 3.5. Injury Risk Models

Unadjusted odd ratios from our binary logistic regression models showed an increase in injury risk of 1.12, 14.2 and 1.01 times with greater experience, male sex, and longer hours worked per week, respectively (Table 4). After adjustment, male ever-miners were still significantly (9.34 times) more likely to get injured than female ever-miners. However, mining experience and total working hours per week were no longer significantly associated with increased injury risk. Neither continuous nor categorical measures of BMI or education were found to be confounding factor or significantly associated with injury risk. 

**Table 4:** Binary logistic regression model predicting odds ratio (OR) for past-year injury in 2011 (N = 69 miners).

Variable	Unadjusted				Adjusted		
	Intercept	OR	95% CI		Intercept	OR	95% CI
Male sex	0.1	14.2 *	4.3–46.7		1.0	9.3 *	2.5–34.4
Total working hours/week	0.7	1.01	1.0–1.02			1.01	1.0–1.02
Mining experience (years)	0.3	1.12 *	1.04–1.2			1.1	1.0–1.2

* *p* < 0.05.

## 4. Discussion

We have documented evidence of a range of occupational hazards and injuries among ASG miners in Ghana's Upper East Region. Participants reported participating in a diverse assortment of mining activities and demonstrated a wide range of mining experience, from days to decades. Demographic factors generally were not predictive of injury risk, though males showed a significantly increased risk of injury, and BMI and smoking status showed a potential (but non-significant) relationship with injury risk. Psychological demands and perceived stress were high among the miners assessed, and training on occupational health and safety issues and use of PPE were rare. The most common injury location was lower limbs (legs and feet), the most common injury causes was falling, and the most common type of injury was a cut or laceration. Logistic regression results suggested that mining experience and hours per week worked were slightly but non-significantly associated with increased injury risk, while male sex showed a significantly increased risk of injury. Age, a possible confounder with mining experience, was not significantly associated with injury risk and thus not included in our regression model. 

We estimated a past-year injury rate of 45.5 injuries per 100 person-years in 2011 and 38.5 injuries per 100 person-years in 2013 among our participants. After adjusting for the number of hours worked in the miner’s primary mining activity in the week preceding the survey, these rates were 42.0 injuries per 100 person-years and 29.9 injuries per 100 person-years, respectively. The differences between these two sets of estimates are relatively small, and it seems likely that the true injury rate lies somewhere in the range between them. These results are consistent with other studies of small-scale mining operations in Africa that have demonstrated elevated injury rates. Approximately 71% of 180 artisanal miners in the D.R.C reported at least one occupational injury in 2009, and an average of two injuries per person per year (total 392 injuries) [19]. This equates to an astoundingly high annual rate of 217.8 injuries per 100 person-years. Rates of non-fatal incidents and illnesses in large-scale mining operations in Africa appear much lower. A multi-year study of 13,924 copper mining workers in Zambia reported 165 total injuries. The estimated injury rate was 0.55 per 100 person-years, respectively, though non-fatal cases in that study were restricted to severe injuries, or injuries requiring medical attention with missing work day(s) [25]. A rate of 0.84 injuries per 100 FTE (approximately 0.84 injuries per 100 person-years) was reported in October 2009 among gold miners in South Africa [26]. Collectively, this evidence supports statements by the International Labor Organization that ASM operations may have several times more non-fatal accidents than large-scale operations [13]. For comparison purposes, non-fatal injury rates in the US in 2011 were 3.5 injuries per 100 FTE (approximately 3.5 injuries per 100 person-years) for all private industries and 2.2 injuries per 100 FTE (approximately 2.2 injuries per 100 person-years) for the mining industry [27]. 

A recent study (also published in this special edition (http://www.mdpi.com/journal/ijerph/special_issues/asgm)) in the Tarkwa mining district in Ghana’s Western Region employed methods similar to ours to profile injuries among both legal and illegal small-scale gold miners. In this study, 404 participants reported 121 injuries severe enough to make them miss days of work in the ten years (2245 person-work years) preceding the survey. This yielded an overall severe injury rate of 5.39 per 100 person years [28]. This severe-injury rate is much lower than the all-injury rate (which could include even injuries requiring only first-aid) in our own study. This is due at least in part to the fact that severe injuries occur much less frequently than minor injuries. However, the injury rate difference may also indicate differences in work conditions between the mines studied by Calys-Tagoe *et al.* and those in our own study. For example, only 12.1% of miners in Calys-Tagoe *et al*.’s study reported ever refusing to work because of safety concerns, compared to over half of the miners in our study [28]. Calys-Tagoe *et al*. identified the most common cause of injury as being hit or struck by an object. The study also found that the most common type of injury was a cut or laceration, which agrees with our own findings. Patterns in body parts injured were also similar between the two studies.

Another study (also published in this special edition (http://www.mdpi.com/journal/ijerph/special_issues/asgm)) on severe injuries from ASGM in Ghana, which examined 72 hospital records from Nkawkaw Hospital in the Eastern Region from between 2006 and 2013, found that when causes of injuries were listed, they were most frequently collapsed mine pits [29]. Fractures (30.5%) and contusions (29.1%), followed by spinal cord injuries (18.0%), were the most common types of injury listed, which is not surprising given the severe type of accident most commonly identified in their study. Collectively, these three studies of ASGM in Ghana—our own and those of Calys-Tagoe *et al*. and Kyeremateng-Amoah and Clarke—indicate that ASG miners have elevated all-injury and severe-injury risk.

Our study also demonstrates substantial psychosocial stressors among ASG miners in Ghana. Male ever-miners had significantly higher psychological job demands scores than did females. For comparison purposes, female ever-miners in our study had an average psychological job demands score that was slightly lower (better) than the average for female workers in the US Quality of Employment Survey (QES) [22], while male ever-miners scored slightly worse than US male workers. The average scores of co-worker support for female and male ever-miners were slightly higher (better) than average scores of female and male workers in the US [22]. PSS scores were higher among female and male ever-miners in our study (average score was approximately one-half of the possible scale, with scores greater than half indicating “high stress”) than among US workers (average score was approximately one-quarter of the full scale), but comparable to scores among low-income and mostly black or multiracial South African adults (about half of the total scale) [30]. Male ever-miners in our study perceived higher stress than females, while the opposite was true among US workers and low-income South African workers [23]. Our results indicate that miners have concerns about their safety; over half of participants in our study had at some point refused work due to safety concerns, and 80% had made complaints about their safety while mining. Our results further suggest that safety training in ASGM communities is inadequate. Only two miners in our study had ever received any form of occupational safety training (and that they were the two miners injured in both 2011 and 2013). Similarly, Calys-Tagoe *et al*. 2015 found that less than a third of the 404 miners surveyed had ever received any mining safety training [28]. Reported PPE use was very low in our study, consistent with findings of other studies of ASG miners in Ghana and sub-Saharan Africa [17,18]. 

Our study has a number of limitations. The primary limitation is its small sample size, especially for the 2013 subsample, which may limit our ability to generalize our results. Limited resources allowed for a sample size of 173 in 2011. Of these, only 22 participants could be identified in 2013, primarily due to the transient nature of miners' lifestyles and lack of mobile phone numbers or other means of contacting former participants in advance to arrange their 2013 interviews. Many miners return to their home villages to farm depending on the season and the weather, and some take multiple-day trips to nearby villages or cities for family or religious events, selling gold, or purchasing or repairing equipment. Future longitudinal studies could mitigate the loss of participants by anticipating a high rate of subject attrition and recruiting a higher-than-necessary number of subjects, collecting detailed contact information (e.g., mobile phone numbers, alternate dwelling locations), and following up more regularly with participants to determine their locations prior to scheduling interviews. It is important to note that even the collection of mobile phone numbers does not guarantee access to subjects, as many mining areas lack mobile phone coverage.

A second limitation relates to the nature of tasks involved in ASGM work, and likely influenced our observed injury outcomes. Mining tasks appear segregated by sex, with women predominantly working in sifting and less frequently in other tasks, and men rarely working in sifting but performing all other tasks. Sifting appears to be one of the least dangerous parts of the mining process, which may help explain why the injury rate for females was so much lower than that for males. While we had data on the duration of each miner’s experience, no data were collected regarding the timing of this experience. Thus, though a miner may have had several years of experience in excavation, for example, it is possible that this experience could have occurred before the period that the questions on injury addressed (the one year prior to the survey). This may partially explain the lack of a significant relationship between mining activity type and injury rates. Further research examining injury differences by gender may benefit from oversampling female miners working in activities other than sifting. 

We also assumed a full-time work schedule (40 hours per week) for our participants for one set of injury risk estimates. Though on average participants reported working nearly full-time (36.9 and 31.0 hours per week in 2011 and 2013, respectively), our measure of hours worked per week was imperfect. The survey asked about the number of hours worked in the week preceding the survey and only in the participant’s primary mining activity, which may not be representative of the entire year preceding the survey, and may underestimate the amount of time spent working if the participant does other mining activities. It is thus unclear that adjusting for number of hours worked per week resulted in a more accurate injury rate.

An additional limitation is the possibility that our study results are biased. We attempted to limit recall bias by restricting our injury recall period to the previous year, but it is possible that some injuries were not reported due to insufficient recall, or that injuries were reported inaccurately. This is likely a larger issue for minor injuries than for major injuries. The healthy worker effect may have caused some injured or ill miners to leave ASGM work and move away from the mining community sampled, but given the relative lack of employment alternatives for individuals involved in ASGM mining, this does not seem likely. Selection bias is likewise a possibility. However, within the community, no households refused to participate in the study for reasons obviously related to health status or other issues related to occupational health or injury, so we feel this is also unlikely. Finally, we excluded from our injury analysis those who had not mined within the last 3 months; due to a survey design flaw, mining injuries in the past year were not accurately recorded for these individuals. This exclusion may have biased our results negatively (*i.e.*, by excluding individuals who worked injury-free as miners) or positively (*i.e.*, by excluding injured miners who had not done mining within the past 3 months). 

## 5. Conclusions 

As one of the first studies to qualitatively and quantitatively assess injuries at ASGM sites in Ghana, the research described here helps address the paucity of occupational health and safety information on artisanal mining in the existing literature. Future studies should consider recording injuries among all community members to determine the proportion related to surrounding mining activities. Adequately funded, larger-scale and longitudinal studies using systematic survey methods—including improved methods of contacting transient miners combined with regular schedule of contact with subjects—or data already available in medical records from hospitals and clinics are needed to further elucidate the causes, nature, severity, and frequency of injuries at ASM sites in Ghana. However, such studies—and, in particular, studies based on medical records—must be designed carefully, as records are likely to contain substantial bias due to geographical and income-related disparities in medical access.

It is also worth noting that injuries may be a significant source of morbidity and mortality not just for miners, but also for non-miners. Anecdotal evidence from participants in our study suggests that non-miners and children have fallen into uncovered and abandoned mine pits, resulting in severe injury and even death. No studies that we are aware of to date have examined the prevalence of non-miner injuries due to ASGM. 

Finally, our study suggests that, despite the relative uncertainty in our injury estimates due to the sample size limitations, the burden of injuries in ASGM communities is quite large. Further research to better characterize injury rates and determinants of injury risk is needed, but there is a clear need for occupational health interventions even in the absence of additional research. Additional resources should be dedicated to reducing the burden of injury and death in ASGM communities; such efforts will likely improve both occupational and public health in these communities.

## Supplementary Material

**Table S1:** Comparison of mining experience between male and female ever-miners in 2011, 2013.

		Female		Male	
		N (%)	Experience (yr)	N (%)	Experience (yr)
**2011**	**Mining Activities**	37 (100)		49 (100)	
	-Ever Excavate	2 (5.4) ^***^	0.9 ± 0.1	42 (85.7)	8.0 ± 6.0
	-Ever Crushed	11(29.7) ^***^	3.5 ± 5.3	42 (85.7)	7.2 ± 6.3
	-Ever Sifted	34 (91.9) ^***^	4.6 ± 5.5 ^*^	16 (32.7)	8.3 ± 6.5
	-Ever Washed	15 (40.5) ^***^	3.0 ± 4.5 ^***^	35 (71.4)	9.5 ± 6.7
	-Ever Amalgamated	20 (54.1) ^*^	2.8 ± 4.2 ^***^	36 (73.5)	9.2 ± 6.7
	-Ever Burned	9 (24.3) ^***^	1.9 ± 2.4 ^**^	28 (57.1)	7.1 ± 5.5
	-Ever Owned/Managed	1 (2.70) ^***^	0	21 (42.9)	5.7 ± 5.7
	**Overall Mining Experience**	-	4.5 ± 5.3 ^***^	-	8.7 ± 6.7
**2013**	**Mining Activities**	8 (100)		9 (100)	
	- Ever Excavate	3 (37.5)	2.7 ± 1.2	7 (77.8)	6.6 ± 6.9
	-Ever Crushed	2 (25.0) ^***^	2.0 ± 0.0	9 (100)	6.1 ± 6.0
	-Ever Sifted	8 (100) ^***^	6.8 ± 5.2	2 (22.2)	8.0 ± 7.1
	-Ever Washed	6 (75.0)	4.7 ± 4.0	7 (77.8)	9.6 ± 6.4
	-Ever Amalgamated	7 (67.5)	4.0 ± 4.0 ^*^	7 (77.8)	10.4 ± 5.6
	-Ever Burned	4 (50.0)	2.5 ± 1.7	5 (55.6)	6.4 ± 6.0
	-Ever Owned/Managed	0 ^*^	0	5 (55.6)	8.8 ± 6.6
	**Overall Mining Experience**	-	6.8 ± 5.2	-	8.5 ± 6.4
**2011**	**Total working hours/week**	27	23.8 ± 33.7	44	45.0 ± 51.7
**2013**	**Total working hours/week**	4	25.9 ± 24.2	8	33.6 ± 32.2

Difference between male and female ever-miners * *p* ≤ 0.05, ** *p* ≤ 0.01 *** *p* ≤ 0.005.
